# Supplementary material for: Acute severe asthma requiring invasive mechanical ventilation in the era of modern resuscitation techniques: A 10-year bicentric retrospective study
Source: PLoS One. 2020 Oct 2;15(10):e0240063. doi: 10.1371/journal.pone.0240063 (PMC7531794; doi:10.1371/journal.pone.0240063)
Supplement: S1 Table — (DOCX) [file pone.0240063.s001.docx]

**S1 Table. Therapeutic management on the day of admission and use of salvage therapies according to in-hospital survival status**

| **Variables** | **All patients**  **(n = 81)** | | **Survivors**  **(n = 69)** | | **Non-survivors**  **(n = 12)** | | ***p*** |
| --- | --- | --- | --- | --- | --- | --- | --- |
|  | DA (n) | Median [IQR]  or n (%) | DA (n) | Median [IQR]  or n (%) | DA (n) | Median [IQR]  or n (%) |  |
| Short-acting beta agonist | 76 | 73 (96%) | 66 | 64 (97%) | 10 | 9 (90%) | 0.35 |
| Short-acting muscarinic antagonist | 76 | 66 (87%) | 66 | 58 (88%) | 10 | 8 (80%) | 0.61 |
| Intravenous short-acting beta agonist | 77 | 6 (8%) | 66 | 6 (9%) | 11 | 0 (0%) | 0.59 |
| Systemic corticosteroid | 77 | 64 (83%) | 66 | 57 (86%) | 11 | 7 (64%) | 0.08 |
| Magnesium sulfate (infusion) | 77 | 52 (68%) | 66 | 45 (68%) | 11 | 7 (64%) | 0.74 |
| Propofol | 80 | 13 (16%) | 68 | 12 (18%) | 12 | 1 (8%) | 0.68 |
| Midazolam | 79 | 53 (67%) | 68 | 48 (71%) | 11 | 5 (46%) | 0.16 |
| Fentanyl or sentinel | 79 | 65 (82%) | 68 | 59 (87%) | 11 | 6 (55%) | **0.02** |
| Continuous neuromuscular blockade | 80 | 52 (65%) | 68 | 46 (68%) | 12 | (50%) | 0.33 |
| Continuous ketamine  Catecholamine infusion | 80 | 21 (26%) | 68 | 19 (28%) | 12 | 2 (17%) | 0.5 |
| Norepinephrine | 78 | 30 (39%) | 68 | 24 (35%) | 10 | 6 (60%) | 0.17 |
| Epinephrine | 78 | 18 (23%) | 68 | 13 (19%) | 10 | 5 (50%) | **0.05** |
| Dobutamine | 79 | 0 (0%) | 68 | 0 (0%) | 11 | 0 (0%) | 1.00 |
| Renal replacement therapy | 81 | 4 (5%) | 69 | 0 (0%) | 12 | 4 (33%) | **<0.01** |
| Fluid administration (mL) | 75 | 1000 [0-1750] | 65 | 1000 [0-1500] | 10 | 750 [0-3500] | 0.85 |
| Fluid balance (mL) | 71 | 550 [130-1650] | 64 | 525 [115-1608] | 7 | 1090 [460-3800] | 0.12 |
| Mechanical ventilator settings |  |  |  |  |  |  |  |
| Tidal volume (mL) | 72 | 405 [375-455] | 63 | 420 [380-475] | 9 | 375 [360-400] | 0.08 |
| Respiratory rate (/min) | 73 | 15 [12-16] | 63 | 15 [12-16] | 10 | 15 [12-18] | 0.59 |
| PEEP (cmH_2_O) | 73 | 2 [0-5] | 63 | 2 [0-5] | 10 | 0 [0-5] | 0.88 |
| Minute ventilation (L/Min) | 73 | 6.0 [5.0-7.1] | 63 | 6.1 [4.9-7.2] | 10 | 6.0 [5.5-6.5] | 0.46 |
| Peak inspiratory pressure (cmH_2_O) | 73 | 50 [38-63] | 63 | 50 [38-63] | 10 | 50 [38-64] | 0.78 |
| FiO_2_ (%) | 67 | 60 [50-100] | 58 | 60 [50-80] | 9 | 100 [70-100] | **<0.01** |
| Salvage therapy  (ECMO, halogenated gas or mepolizumab) | 81 | 7 (9%) | 69 | 5 (7%) | 12 | 2 (17%) | 0.28 |

DA : data available; IQR : interquartile range; PEEP : positive end-expiratory pressure.
