# Supplementary material for: Acute severe asthma requiring invasive mechanical ventilation in the era of modern resuscitation techniques: A 10-year bicentric retrospective study
Source: PLoS One. 2020 Oct 2;15(10):e0240063. doi: 10.1371/journal.pone.0240063 (PMC7531794; doi:10.1371/journal.pone.0240063)
Supplement: S2 Table — (DOCX) [file pone.0240063.s002.docx]

**S2 Table. Characteristics and outcome of the 7 patients who received salvage therapies**

| **Year** | **Age** | **Gender** | **Salvage Therapy** | **Indication** | **SAPS II** | **SOFA** | **CA on day of admission** | **Status** |
| --- | --- | --- | --- | --- | --- | --- | --- | --- |
| 2012 | 69 | Female | Halogenated gas | Refractory bronchospasm | 40 | 3 | no | alive |
| 2012 | 24 | Female | vv-ECMO  Conversion to va-ECMO (day 2) | Respiratory acidosis  Cardiogenic shock post-CA | 87 | 11 | **yes** | **dead** |
| 2013 | 38 | Female | vv-ECMO | Cardiac tamponade due to gas trapping | 43 | 7 | no | alive |
| 2013 | 42 | Female | vv-ECMO | Tension pneumothorax | 72 | 8 | no | alive |
| 2016 | 39 | Female | va-ECMO | Refractory bronchospasm & associated influenza myocarditis | 65 | 6 | no | alive |
| 2017 | 61 | Female | vv-ECMO  Mepolizumab (anti-IL5) | Respiratory acidosis  Refractory bronchospasm | 24 | 7 | no | alive |
| 2017 | 57 | Female | va-ECMO | Cardiogenic shock post-CA | 54 | 10 | **yes** | **dead** |

SAPS 2 : simplified acute physiology score 2; SOFA : sequential organ failure assessment; CA : cardiac arrest; vv-ECMO : venovenous extracorporeal membrane oxygenation; va-ECMO : venoarterial extracorporeal membrane oxygenation.
