# Supplementary material for: Acute severe asthma requiring invasive mechanical ventilation in the era of modern resuscitation techniques: A 10-year bicentric retrospective study
Source: PLoS One. 2020 Oct 2;15(10):e0240063. doi: 10.1371/journal.pone.0240063 (PMC7531794; doi:10.1371/journal.pone.0240063)
Supplement: S3 Table — (DOCX) [file pone.0240063.s003.docx]

**S3 Table. Complications and outcome according to in-hospital survival status**

| **Variables** | **All patients**  **(n = 81)** | | **Survivors**  **(n = 69)** | | **Non-survivors**  **(n = 12)** | | ***p*** |
| --- | --- | --- | --- | --- | --- | --- | --- |
|  | DA (n) | Median [IQR]  or n (%) | DA (n) | Median [IQR]  or n (%) | DA (n) | Median [IQR]  or n (%) |  |
| Any type of complications | 81 | 37 (46%) | 69 | 25 (36%) | 12 | 12 (100%) | **<0.01** |
| Pulmonary barotrauma | 81 | 4 (5%) | 69 | 3 (4%) | 12 | 1 (8%) | 0.48 |
| Pneumothorax | 81 | 3 (4%) | 69 | 2 (3%) | 12 | 1 (8%) | 0.39 |
| Pneumomediastinum | 81 | 1 (1%) | 69 | 0 (0%) | 12 | 1 (8%) | 0.15 |
| Subcutaneous emphysema | 81 | 3 (4%) | 69 | 2 (3%) | 12 | 1 (8%) | 0.39 |
| Ventilator associated pneumonia | 81 | 20 (25%) | 69 | 16 (23%) | 12 | 4 (33%) | 0.48 |
| Other ICU-acquired infections | 81 | 12 (15%) | 69 | 9 (13%) | 12 | 3 (25%) | 0.37 |
| ICU-acquired weakness | 81 | 5 (6%) | 69 | 5 (7%) | 12 | 0 (0%) | 1.00 |
| Severe cardiac arrhythmia | 81 | 5 (6%) | 69 | 4 (6%) | 12 | 1 (8%) | 0.56 |
| Myocardial ischemia | 81 | 5 (6%) | 69 | 3 (4%) | 12 | 2 (17%) | 0.16 |
| Shock related to ASA | 81 | 5 (6%) | 69 | 4 (6%) | 12 | 1 (8%) | 0.56 |
| Severe atelectasis | 81 | 6 (7%) | 69 | 6 (9%) | 12 | 0 (0%) | 0.58 |
| Tracheostomy | 81 | 6 (7%) | 69 | 5 (7%) | 12 | 1 (8%) | 1.00 |
| ICU length of stay (days) | 81 | 7 [4-12] | 69 | 7 [5-12] | 12 | 4 [2-13] | 0.08 |
| Hospital length of stay (days) | 81 | 13 [8-17] | 69 | 13 [10-17] | 12 | 4 [2-13] | **<0.01** |

DA : data available; IQR : interquartile range; ICU : intensive care unit; ASA : acute severe asthma.
