# Supplementary material for: Acute severe asthma requiring invasive mechanical ventilation in the era of modern resuscitation techniques: A 10-year bicentric retrospective study
Source: PLoS One. 2020 Oct 2;15(10):e0240063. doi: 10.1371/journal.pone.0240063 (PMC7531794; doi:10.1371/journal.pone.0240063)
Supplement: S4 Table — (DOCX) [file pone.0240063.s004.docx]

**S4 Table. Characteristics of the 12 patients who died in hospital according to whether or not they presented a cardiac arrest on the day of admission**

| **Patients who presented a CA on the day of admission** | | | | | | |
| --- | --- | --- | --- | --- | --- | --- |
| **Year** | **LOS in hospital** | **Age** | **Male gender** | **Lactate on**  **day of admission** | **Decompensation risk factor** | **Evolution and cause of death** |
| 2008 | 2 | 47 | 0 | 10.3 | Unknown | CA at home  => Refractory shock post-CA with MOF |
| 2010 | 4 | 48 | 0 | MD | Unknown | CA at home  => Brain death |
| 2010 | 16 | 56 | 0 | 15 | Unknown | CA in the emergency department  => Post-anoxic encephalopathy |
| 2012 | 4 | 58 | 0 | 1.7 | Unknown | CA at home  => Brain death |
| 2012 | 4 | 24 | 0 | 9 | Unknown | CA while driving to the hospital  vv-ECMO for refractory bronchospasm  Conversion to va-ECMO for cardiogenic shock post-CA  => Brain death |
| 2016 | 16 | 67 | 0 | 9.6 | Unknown | CA in the emergency department  Slight improvement at first  Candidemia  => Refractory shock due to Candidemia with MOF |
| 2017 | 2 | 30 | 0 | 15 | Unknown | CA at home  => Refractory shock post-CA with MOF |
| 2017 | 2 | 36 | 1 | 22 | Unknown | CA during transportation to the emergency department  => Refractory shock post-CA with MOF  => Post-anoxic encephalopathy |
| 2017 | 2 | 57 | 0 | 1.7  (before CA) | Pneumonia  (unknown agent) | CA on arrival in ICU  va-ECMO for cardiogenic shock post-CA  => Refractory shock post-CA with MOF  => Acute mesenteric ischemia |

| **Patients who did not presented a CA on the day of admission** | | | | | | |
| --- | --- | --- | --- | --- | --- | --- |
| **Year** | **LOS in ICU** | **Age** | **Male gender** | **Lactate on**  **day of admission** | **Decompensation risk factor** | **Evolution and cause of death** |
| 2008 | 45 | 72 | 0 | MD | Pneumococcal pneumonia | VAP (Serratia marcesens)  Difficult weaning from mechanical ventilation  Tracheostomy  Discharge to medical ward with tracheostomy  => Hypoxic CA in medical ward (tracheostomy mucus plug) |
| 2013 | 1 | 82 | 0 | 4 | Unknown | Withholding of life support due to age and comorbidities  => Severe shock with MOF |
| 2016 | 10 | 79 | 0 | 2.9 | Respiratory syncytial virus pneumonia | VAP (*Pseudomonas aeruginosa* and *Klebsiella pneumoniae*)  => Refractory shock due to VAP with MOF |

CA : cardiac arrest; LOS : length of stay; ICU : intensive care unit; MOF : multiple organ failure; MD : missing data; vv-ECMO : venovenous extracorporeal membrane oxygenation; va-ECMO : venoarterial extracorporeal membrane oxygenation; VAP: ventilator-associated pneumonia.
